# Ceramide changes in abdominal subcutaneous and visceral adipose tissue among diabetic and nondiabetic patients

**DOI:** 10.1111/1753-0407.13262

**Published:** 2022-04-25

**Authors:** Michelle Brusatori, Michael H. Wood, Stephanie C. Tucker, Krishna Rao Maddipati, S. Kiran Koya, Gregory W. Auner, Kenneth V. Honn, Berhane Seyoum

**Affiliations:** ^1^ Michael and Marian Ilitch Department of Surgery School of Medicine, Wayne State University Detroit Michigan USA; ^2^ Smart Sensors and Integrated Microsystems Program Wayne State University Detroit Michigan USA; ^3^ Harper Bariatric Medicine Institute Harper University Hospital, Detroit Medical Center Detroit Michigan USA; ^4^ Department of Pathology Bioactive Lipids Research Program and Lipidomics Core Facility, Wayne State University Detroit Michigan USA; ^5^ Division of Endocrinology Wayne State University, School of Medicine Detroit Michigan USA

**Keywords:** adipose tissue, adiposity, ceramides, diabetes, gender, insulin resistance, 神经酰胺, 糖尿病, 脂肪组织, 肥胖, 胰岛素抵抗, 性别

## Abstract

**Background:**

This study profiles ceramides extracted from visceral and subcutaneous adipose tissue of human subjects by liquid chromatography‐mass spectrometry to determine a correlation with status of diabetes and gender.

**Methods:**

Samples of visceral and abdominal wall subcutaneous adipose tissue (n = 36 and n = 31, respectively) were taken during laparoscopic surgery from 36 patients (14 nondiabetic, 22 diabetic and prediabetic) undergoing bariatric surgery with a body mass index (BMI) >35 kg/m^2^ with ≥1 existing comorbidity or BMI ≥40 kg/m^2^. Sphingolipids were extracted and analyzed using liquid chromatography‐mass spectrometry.

**Results:**

After logarithm 2 conversion, paired analysis of visceral to subcutaneous tissue showed differential accumulation of Cer(d18:1/16:0), Cer(d18:1/18:0), and Cer(d18:1/24:1) in visceral tissue of prediabetic/diabetic female subjects, but not in males. Within‐tissue analysis showed higher mean levels of ceramide species linked to insulin resistance, such as Cer(d18:1/18:0) and Cer(d18:1/16:0), in visceral tissue of prediabetic/diabetic patients compared with nondiabetic subjects and higher content of Cer(d18:1/14:0) in subcutaneous tissue of insulin‐resistant female patients compared with prediabetic/diabetic males. Statistically significant differences in mean levels of ceramide species between insulin‐resistant African American and insulin‐resistant Caucasian patients were not evident in visceral or subcutaneous tissue.

**Conclusions:**

Analysis of ceramides is important for developing a better understanding of biological processes underlying type 2 diabetes, metabolic syndrome, and obesity. Knowledge of the accumulated ceramides/dihydroceramides may reflect on the prelipolytic state that leads the lipotoxic phase of insulin resistance and may shed light on the predisposition to insulin resistance by gender.

## Graphical abstract

- The gender‐specific role of sphingolipids in obesity‐related metabolic dysfunctions is unclear. The aim of this study is to identify ceramide species by liquid chromatography‐mass spectrometry that accumulate in central fat in relation to status of diabetes and gender for subjects with a body mass index ≥40 kg/m^2^ or > 35 kg/m^2^ with comorbidity.
- The results showed significantly increased ceramide levels in visceral tissue of patients with diabetes and prediabetes and unveiled several ceramide species that are differentially expressed in insulin‐resistant females compared to males.

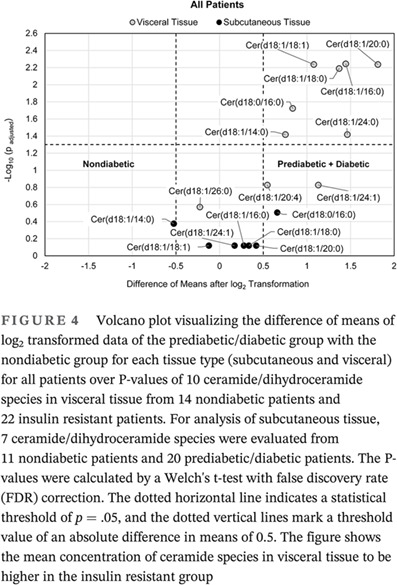

## 摘要

**目的:**

采用液相色谱‐质谱分析从受试者内脏和皮下脂肪组织中提取的神经酰胺, 以确定神经酰胺与糖尿病状态和性别的相关性。

**方法:**

在腹腔镜手术中采集36例内脏和31例腹壁皮下脂肪组织标本, 这些标本来自36位患者, 其中14位非糖尿病, 22例糖尿病及糖尿病前期患者, 体重指数>35 kg/m^2^并有超过1种合并症, 或体重指数≥40 kg/m^2^。采用液相色谱‐质谱联用技术对鞘磷脂进行提取和分析。

**结果:**

Log2转换后, 内脏和皮下组织的配对分析显示Cer(d18:1/16:0), Cer(d18:1/18:0)和Cer(d18:1/24:1)在糖尿病前期/糖尿病女性受试者的内脏组织中有不同程度的蓄积, 而男性则没有。组织内分析显示, 糖尿病前期/糖尿病患者内脏组织中与胰岛素抵抗相关的神经酰胺类物质如Cer(d18:1/18:0)和Cer(d18:1/16:0)的平均水平高于非糖尿病患者, 而胰岛素抵抗女性患者皮下组织中Cer (d18:1/14:0) 的含量高于糖尿病前期/糖尿病男性患者。在胰岛素抵抗的非裔美国人和胰岛素抵抗的高加索人之间, 神经酰胺的平均水平在内脏或皮下组织中没有明显的统计学差异。

**结论:**

神经酰胺的分析对于更好地了解2型糖尿病, 代谢综合征和肥胖的生物学过程很重要。对积累的神经酰胺/二氢神经酰胺的了解可以反映导致胰岛素抵抗的脂毒性阶段的脂解前状态, 并可能揭示性别对胰岛素抵抗的易感性。

## 1 INTRODUCTION

Studies using cultured cells, animal models, or human subjects have demonstrated that ceramides are the key intermediate factors directly involved in inducing insulin resistance (IR).^1^, ^2^ Ceramides act to inhibit systemic glucose uptake and glycogen synthesis.^3^, ^4^ The presence of excess fatty acids and oxidative stress is one plausible factor that could be the reason for the IR, as prolonged exposure of adipocyte and myocyte cell cultures to palmitic acid caused a significant increase in ceramide production and inhibition of protein kinase B (Akt) signaling.^5^, ^6^ Inhibition of enzymes involved in ceramide biosynthesis by pharmacologic agents or small interfering RNA showed a significant reduction of IR.^7^, ^8^, ^9^ On the other hand, overexpression of acid ceramidase that metabolizes ceramides has been shown to reduce ceramide levels and significantly improve insulin signaling. Furthermore, it has been reported that consumption of a high‐fat diet increases the production of ceramide synthase—the enzyme that synthesizes ceramides.^10^ Similarly, intravenous infusion of lipid emulsion using the euglycemic‐hyperinsulinemic clamp technique has also been shown to increase muscle ceramide content and induce IR.^11^, ^12^ Ceramide accumulation is reportedly more than 2‐fold higher among subjects with IR when compared with insulin‐sensitive subjects.^12^ In addition to improving glycemic control in patients with diabetes, physical exercise also decreases muscle ceramide content among humans.^13^ Because of its significance, some researchers have suggested the potential therapeutic effects of ceramide synthesis inhibitors and activators of ceramide degradation to treat IR.^1^

The exact mechanism of how ceramides induce IR is unclear. However, it has been speculated that ceramides interfere in the post‐receptor signaling pathways. Ceramides appear to inhibit insulin receptor substrate by phosphorylating several of its serine residues.^14^ Ceramides also inhibit phosphorylation of Akt.^15^ High levels of ceramides have been reported in patients with IR and type 2 diabetes.^16^, ^17^ Some of the plausible mechanisms for high concentration of ceramides to induce IR are by reducing peripheral glucose uptake, inactivating Akt,^18^, ^19^ and inducing inflammatory milieu via activating nuclear factor kappa B‐tumor necrosis factor alpha (TNF‐α).^20^ To further worsen the accumulation of ceramides in the tissue, TNF‐α, in turn, activates sphingomyelinase, the plasma membrane‐bound enzyme that hydrolyzes sphingomyelin to form ceramides that eventually adds to the worsening of IR. Thus, ceramides are bioactive lipids that play a significant role in creating IR in diabetic subjects. These substances appear to link excess dietary fat intake and cytokine‐induced inflammation in tissues. Human studies indicate an association of adipose tissue ceramide content with obesity and metabolic syndrome.^21^, ^22^, ^23^, ^24^, ^25^ In a small cohort study, Choromańska et al. found a link between metabolic syndrome in European women with morbid obesity and ceramide content in visceral adipose tissue.^21^ Blachnio‐Zabielska et al. and Turpin et al. found in small cohorts elevated levels of ceramides in subcutaneous and white adipose tissue, respectively, in obese subjects compared with lean controls.^22^, ^23^ Chaurasia et al. and Kolak et al. found in small cohorts elevated levels of ceramides in adipose tissue of insulin‐resistant subjects of respective Asian and Finish descent, independent of obesity.^24^, ^25^, ^26^ While ceramides are implicated in metabolic syndrome, the distribution of ceramide/dihydroceramide species between subcutaneous and visceral adipose tissue in relation to status of diabetes and gender has not been thoroughly investigated. Subcutaneous and visceral adipose tissue differ in their anatomical, metabolic, and inflammatory characteristics,^27^, ^28^, ^29^ with visceral adiposity being strongly associated with metabolic disease.^28^ Data indicate sex differences in the accumulation of adipose tissue and in the regulation of adipose tissue metabolism.^30^, ^31^ This study sheds light on the ceramide lipidomic profile of abdominal wall subcutaneous and visceral adipose tissue in relation to the status of diabetes and gender for American patients with similar body mass index (BMI) (≥40 kg/m^2^ or > 35 kg/m^2^ with comorbidity).

## 2 METHODS

### 2.1 Patient selection

We included a total of 36 patients that had undergone bariatric surgery at Harper Bariatric Medicine Institute, Harper University Hospital, Detroit, Michigan. All participants provided written informed consent and the Wayne State University Institutional Review Board approved the protocol. Inclusion criteria for bariatric surgery were according to the National Institutes of Health (NIH) guidelines (age > 18 years, BMI >35 kg/m^2^ with ≥1 existing comorbidity or BMI ≥40 kg/m^2^ and a history of prolonged previous attempts of weight loss by other means).^32^ During laparoscopic surgery, over 200 mg of adipose fat tissue were collected from the visceral fat and abdominal wall subcutaneous adipose tissue from all participants. The samples were immediately cooled on ice and frozen in liquid nitrogen within 10 to 15 minutes. The frozen samples were stored in a −80°C freezer until processed.

The mean age of patients was 41.1 +/− 12.1 years. The number of female patients was greater than the number of males (8 males vs 28 females, *P* < .05), and the number of African American (AA) patients was greater than the number of Caucasian patients (30 AA vs 6, *p* < .05). Of the 36 patients, 14 were nondiabetics, 10 were diabetic, and 12 were prediabetic. For purpose of analysis, patients were grouped into two categories based on diabetic status. One group consisted of nondiabetic patients, while the other group consisted of prediabetic/diabetic patients. Table 1 presents clinical data by patient group.

**TABLE 1:** Clinical data by patient group (mean +/− SD)

Clinical data	Nondiabetic (n = 14)	Prediabetic/diabetic (n = 22)
HbA1c (%)	5.4 +/− 0.2	6.4 +/− 0.8*
Glucose (ng/dL)	97.0 +/− 20.3	106.1 +/− 26.2
Race	13 AA, 1 W	17 AA, 5 W
Age	37.4 +/− 10.5	42.9 +/− 12.5
Gender	13 F, 1 M	15 F, 7 M
BMI (kg/m^2^)	47.8 +/− 10.1	49.8 +/− 6.5
Cholesterol (mg/dL)	194.2 +/− 35.3	191.7 +/− 39.4
HDL (mg/dL)	58.1 +/− 21.8	46.3 +/− 13.2
LDL (mg/dL)	121.8 +/− 23.3	112.6 +/− 26.0
TG (mg/dL)	132.0 +/− 61.6	144.8 +/− 101.7
Systolic BP (mm Hg)	126.7 +/− 15.6	124.1 +/− 15.4
Diastolic BP (mm Hg)	77.0 +/− 11.0	78.1 +/− 8.7

Abbreviations: AA, African American; BMI, body mass index; BP, blood pressure; F, female; HbA1c, glycosylated hemoglobin; HDL, high‐density lipoprotein; LDL, low‐density lipoprotein; M, male; TG, triglycerides; W, White.

* *P* < .05.

Mean preoperative glycosylated hemoglobin levels were assessed by a two‐tailed Welch *t* test and were found to be significantly higher in the prediabetic/diabetic group compared to the nondiabetic group (*p* < .05, t = 5.7). While mean glucose levels, BMI, high‐density lipoproteins (HDL), low‐density lipoproteins (LDL), triglycerides (TG), and systolic and diastolic blood pressure did not statistically differ between the two groups.

### 2.2 Liquid chromatography‐mass spectrometry

Liquid chromatography‐mass spectrometry (LC‐MS) was performed at the Lipidomics Core Facility of the Pathology Department at Wayne State University. Lipids were extracted from visceral and subcutaneous adipose tissue samples, and sphingolipids were analyzed following published methods.^33^

#### 2.2.1 Tissue samples

To prepare tissue homogenate, frozen tissue samples were kept at a ratio of tissue weight to buffer volume as 10% (w/v). Frozen tissue was homogenized (3‐4 times) with ice‐cold buffer (0.25 M sucrose, 25 mM KCl, 50 mM Tris, and 0.5 mM EDTA, pH 7.4). The amount of tissue homogenate for analysis was established through S protein concentration measurement (Bio‐Rad protein assay).

Tissue homogenates were fortified with 50 μL of internal standard (IS) solution followed by the addition of 2 mL of extraction mixture (iso‐propanol:water:ethyl acetate, 30:10:60; v:v:v), vortexed and sonicated three times for 30 seconds, and centrifuged for 10 minutes at 4000 rpm. The supernatant was transferred to a new vial and the sample re‐extracted. The supernatants from the extracts were combined (4 mL total).

#### 2.2.2 LC‐MS instrumentation and procedure

Briefly, the tissue homogenates were supplemented with 10 ng of ceramide IS, Cer(d18:1/17:0) (Avante Polar Lipids), followed by extraction with isopropanol‐water‐ethyl acetate (3:1:6 v/v) as described. The extracts were dried and reconstituted in methanol containing 1 mM ammonium formate and 0.2% formic acid. High‐performance liquid chromatography (HPLC) was performed on a Prominence XR system (Shimadzu) using Targa C8 (5μ, 2.1x20 mm, Higgins Analytical) column. The mobile phase consisted of a gradient between A: methanol‐water‐ammonium formate‐formic acid (5:95:1 mM:0.2 v/v) and B: methanol‐ammonium formate‐formic acid (100:2 mM:0.2 v/v). The gradient program with respect to the composition of B was as follows: 0 minutes, 35%; 2 minutes, 100%; and 5 minutes, 100%. The flow rate was 0.5 mL/minute. The HPLC eluate was directly introduced to the electrospray ionization source of a QTRAP5500 mass analyzer (ABSCIEX) in the positive ion mode with the following conditions: curtain gas, 35 psi; GS1, 45 psi; GS2, 45 psi; temperature, 650°C; ion spray voltage, 5500 V; collision gas, low; declustering potential, 100 V; and entrance potential, 10 V. The eluate was monitored by the multiple reaction monitoring (MRM) method to detect unique molecular ion‐daughter ion combinations for each of the ceramides following m/z 264 as daughter ion with 100 msec dwell time for each transition. Optimized collisional energies (16‐28 eV) and collision cell exit potentials (10 V) were used for each MRM transition. The data were collected using the Analyst 1.6.2 software, and the MRM transition chromatograms were quantitated by MultiQuant software (both from ABSCIEX). Ceramides Cer(d18:1/14:0), Cer(d18:1/16:0), Cer(d18:1/18:0), Cer(d18:1/18:1), Cer(d18:1/20:0), Cer(d18:1/20:4), Cer(d18:1/24:0), Cer(d18:1/24:1), Cer(d18:1/26:0), and dihydroceramide DhCer(d18:0/16:0) were quantified against Cer(d18:1/17:0) IS signal. Each ceramide identity was ascertained by matching retention times of the standards (Avanti Polar Lipids). The method has a detection limit of 0.1 pg and quantitation limit of 1.5 pg on the column.

### 2.3 Statistical analysis

For each patient category (all patients, female, male, AA, and Caucasian), the mean concentration +/− SD of the original data and of the logarithm 2 (log_2_)‐transformed concentration of each ceramide/dihydroceramide species evaluated in this work is presented in Supplementary Tables S1 and S2. Variables with less than 37% missingness, within and between tissue type, for each subgroup (nondiabetic, prediabetic/diabetic, male, female, AA, and Caucasian) were used in the analysis.

#### 2.3.1 Paired analysis

After a log_2_ conversion, ceramide/dihydroceramide species in paired visceral to subcutaneous tissue samples were visualized with a volcano plot. The thresholds for log_2_(fold‐change) were set at −0.5 and 0.5 (indicating a 1.4‐fold restriction of means), and the threshold for *P* values was set at .05. *P* values were calculated by a paired Student *t* test with false discovery rate (FDR) correction in the Benjamini‐Hochberg procedure.

#### 2.3.2 Within‐tissue analysis

After a log_2_ conversion, the difference of means of ceramide species of the prediabetic/diabetic group with the nondiabetic group for each tissue type (subcutaneous and visceral) was visualized with a volcano plot. The thresholds for the difference of means, Z_1_‐Z_2_, were set at −0.5 and 0.5 (indicating a 1.4‐fold restriction of medians), and the threshold for *P* values was set at .05. *P* values were calculated by a Welch *t* test with FDR correction in the Benjamini‐Hochberg procedure. Ceramide levels were not adjusted for age, sex, and race/ethnicity for both paired and within‐tissue analysis. Outliers were detected with a Tukey fence (k = 1.5) and excluded from the analysis. The Shapiro‐Wilk test was used to assess normality.

Categorical variables such as sex and race are reported as the frequency of the study population. A chi‐square analysis was performed to determine if the frequency of members in the study population was significantly different (*p* < .05).

## 3 RESULTS

### 3.1 Lipid analysis

Lipids were analyzed to determine a correlation with the status of diabetes. For analysis, patients were grouped into two categories. One group consisted of nondiabetic patients, while the other group consisted of insulin‐resistant (combined prediabetic/diabetic) patients. Total cholesterol, HDL, LDL, and TG levels were subjected to a Welch *t* test. Statistically significant differences in mean levels between nondiabetic and insulin‐resistant patients were not evident.

### 3.2 Ceramides: Visceral to subcutaneous adipose tissue

**FIGURE 1:**
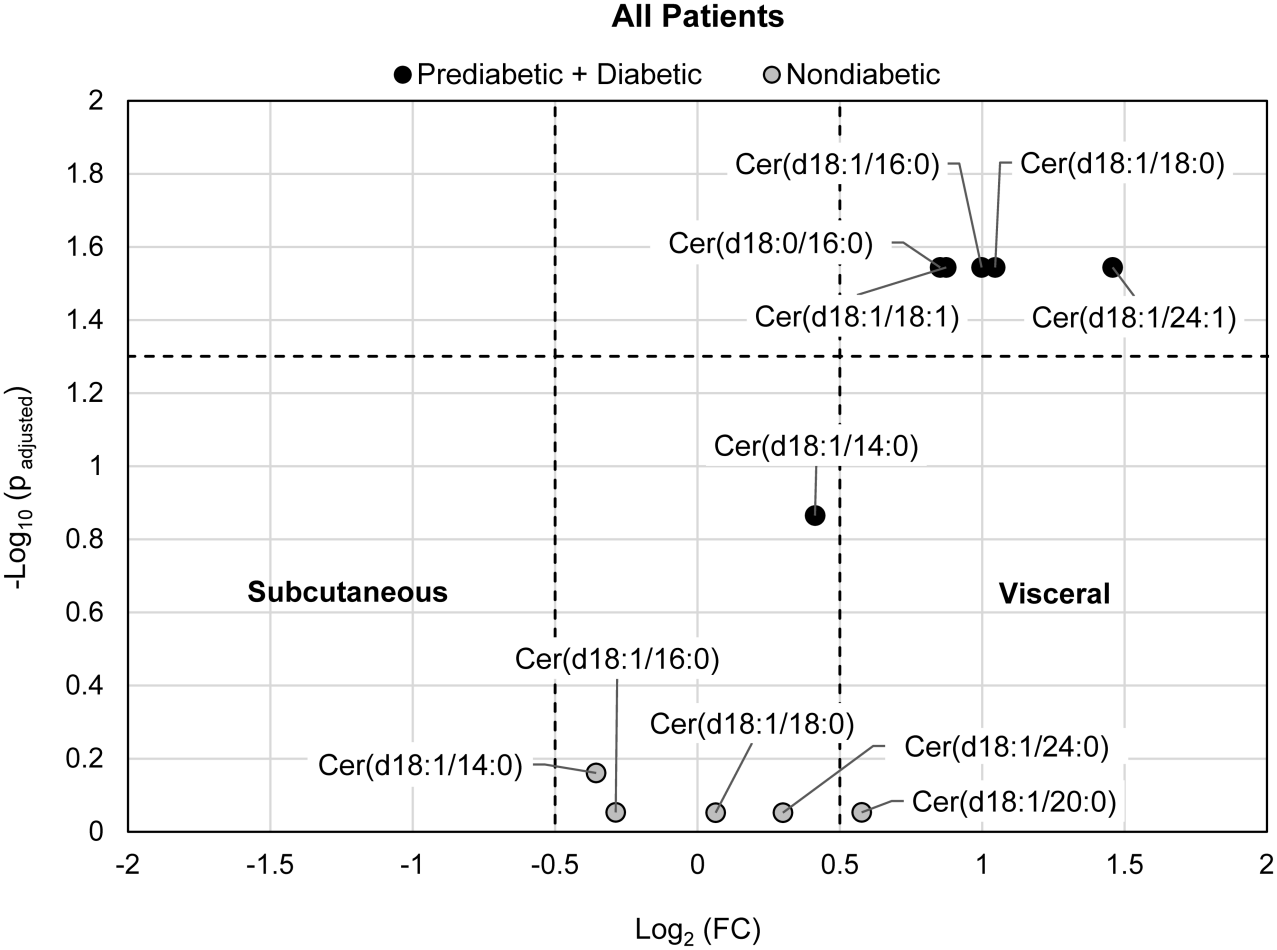
Volcano plot visualizing the fold change (visceral to subcutaneous adipose tissue) over the P‐value of 5 ceramides/dihydroceramides from 11 pairs of adipose tissues from nondiabetic patients and 6 ceramides/dihydroceramides from 20 pairs of adipose tissue from a combined group of prediabetic + diabetic patients. The P‐value of each ceramide species was calculated by paired Student's *t*‐test with false discovery rate (FDR) correction. The dotted horizontal line indicates a statistical threshold of *p* = .05, and the dotted vertical lines between −0.5 and 0.5 log_2_(fold change) indicate a 1.4‐fold restriction threshold. The figure shows that ceramide species are more concentrated in visceral tissue of the insulin resistant group

**FIGURE 2:**
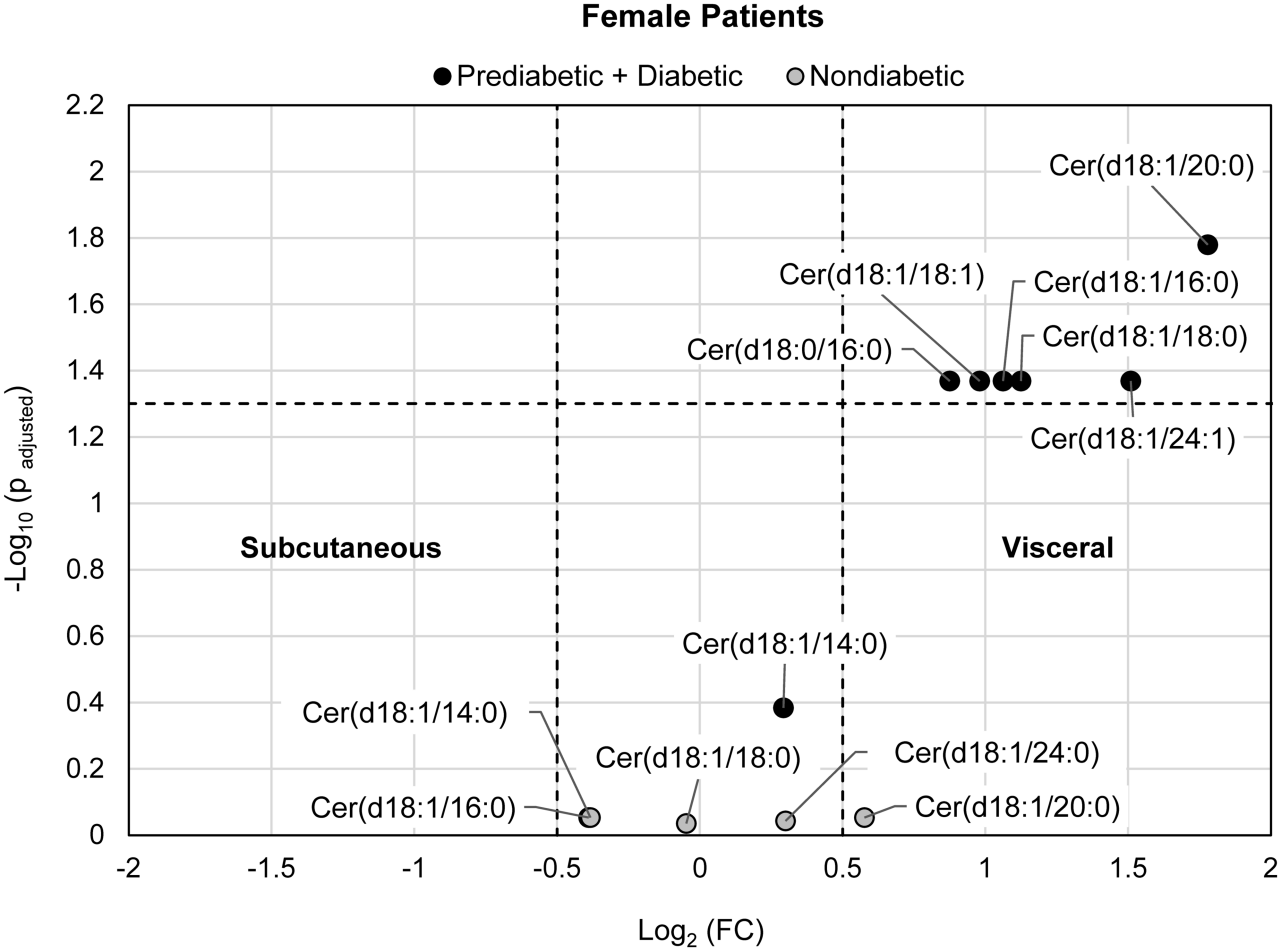
Volcano plot visualizing the fold change (visceral to subcutaneous adipose tissue) over the P‐value of 5 ceramides/dihydroceramides from 10 pairs of adipose tissues from female nondiabetic patients and 7 ceramides/dihydroceramides from 14 pairs of adipose tissue from a combined group of prediabetic + diabetic female patients. The P‐value of each ceramide species was calculated by paired Student's *t*‐test with false discovery rate (FDR) correction. The dotted horizontal line indicates a statistical threshold of *p* = .05, and the dotted vertical lines between −0.5 and 0.5 log_2_(fold change) indicate a 1.4‐fold restriction threshold. The figure shows that ceramide species are more concentrated in visceral tissue of the insulin resistant group

**FIGURE 3:**
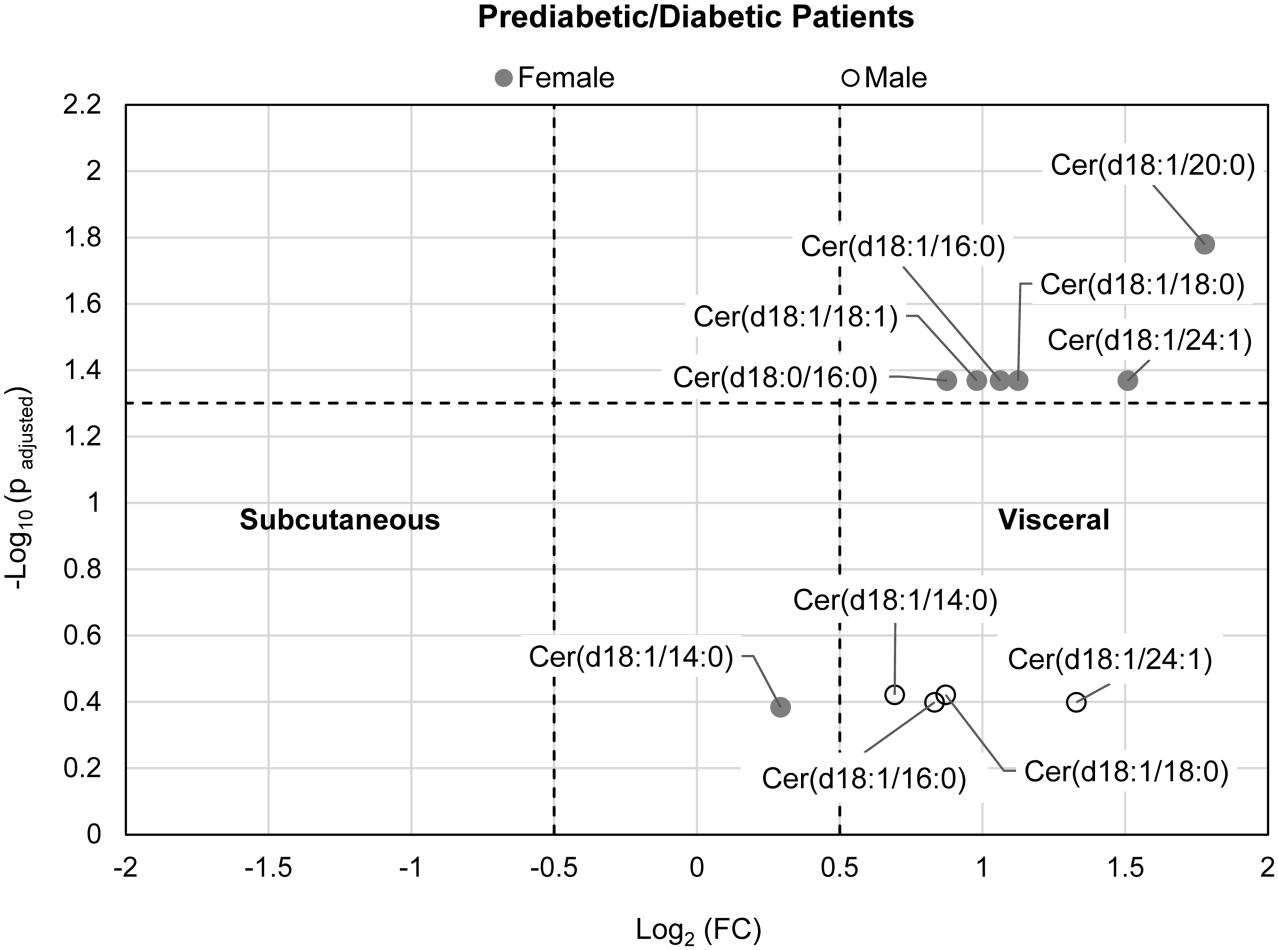
Volcano plot visualizing the fold change (visceral to subcutaneous adipose tissue) over the P‐value of 4 ceramides/dihydroceramides from 6 pairs of adipose tissues from male prediabetic/diabetic patients and 7 ceramides/dihydroceramides from 14 pairs of adipose tissue from a combined group of prediabetic + diabetic female patients. The P‐value of each ceramide species was calculated by paired Student's t‐test with false discovery rate (FDR) correction. The dotted horizontal line indicates a statistical threshold of *p* = .05, and the dotted vertical lines between −0.5 and 0.5 log_2_(fold change) indicate a 1.4‐fold restriction threshold. The figure shows that ceramide species common to both male and female patients are more concentrated in visceral tissue of female subjects

LC‐MS was performed to profile ceramide/dihydroceramide levels in visceral and subcutaneous adipose tissue of patients with BMI ≥40 kg/m^2^ or > 35 kg/m^2^ with comorbidity. Of the 36 patients in the study, 31 had subcutaneous with corresponding visceral tissue samples taken. Separate paired analyses of nondiabetic and insulin‐resistant patients were conducted. The log_2_ fold change (FC) of visceral to subcutaneous tissue over respective *P* values of ceramides is shown in the volcano plots in Figures 1, 2, 3 for all patients (combined group of male and female subjects), female patients only, as well as separate paired analysis by gender, respectively.

For all patients, five out of six ceramide/dihydroceramide species evaluated in the insulin‐resistant group consisting of 20 pairs of adipose tissue from 14 female subjects and 6 male subjects were differentially accumulated in visceral tissue (*P*_adjusted_ < .05, log_2_FC > 0.5). While a significant differential abundance of each of the five ceramide/dihydroceramide species evaluated in the nondiabetic group (from 11 pairs of adipose tissues from 10 female subjects and 1 male subject) was not evident (Figure 1).

Since the nondiabetic group consisted of 10 female subjects and 1 male subject, an analysis of only female patients was performed (with 14 female patients in the insulin‐resistant group). Six of seven species evaluated in the insulin‐resistant group were differentially accumulated in visceral tissue (*P*_adjusted_ < .05, log_2_FC > 0.5). While a significant differential accumulation of each of the five ceramide/dihydroceramide species evaluated in the nondiabetic group was not indicated (Figure 2 **)**.

Paired analyses of visceral to subcutaneous tissue for insulin‐resistant male subjects and insulin‐resistant female subjects are shown in Figure 3. Due to the restriction of missing values, four ceramide species for male patients (n = 6) were evaluated as opposed to seven ceramide/dihydroceramide species for the female group (n = 14). Of the ceramides in common, Cer(d18:1/16:0), Cer(d18:1/24:1), and Cer(d18.1/18:0) showed statistically significant differential accumulation in visceral tissue of insulin‐resistant female patients but not in insulin‐resistant male subjects.

### 3.3 Ceramides between patient groups: Visceral to visceral and subcutaneous to subcutaneous adipose tissue

**FIGURE 4:**
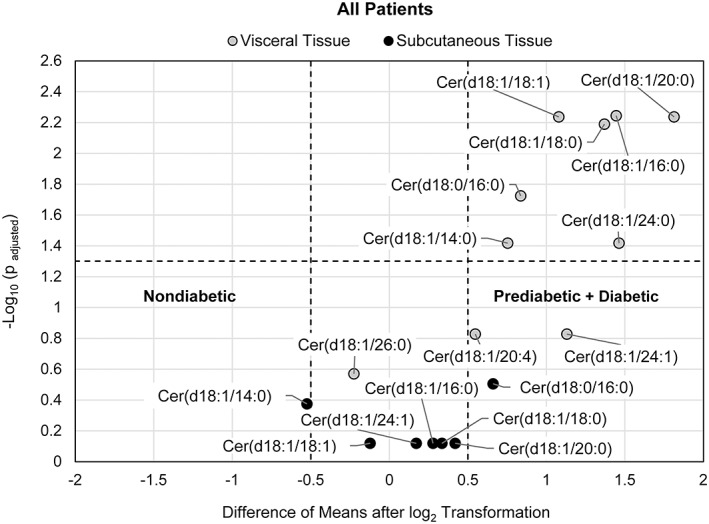
Volcano plot visualizing the difference of means of log_2_ transformed data of the prediabetic/diabetic group with the nondiabetic group for each tissue type (subcutaneous and visceral) for all patients over P‐values of 10 ceramide/dihydroceramide species in visceral tissue from 14 nondiabetic patients and 22 insulin resistant patients. For analysis of subcutaneous tissue, 7 ceramide/dihydroceramide species were evaluated from 11 nondiabetic patients and 20 prediabetic/diabetic patients. The P‐values were calculated by a Welch's t‐test with false discovery rate (FDR) correction. The dotted horizontal line indicates a statistical threshold of *p* = .05, and the dotted vertical lines mark a threshold value of an absolute difference in means of 0.5. The figure shows the mean concentration of ceramide species in visceral tissue to be higher in the insulin resistant group

**FIGURE 5:**
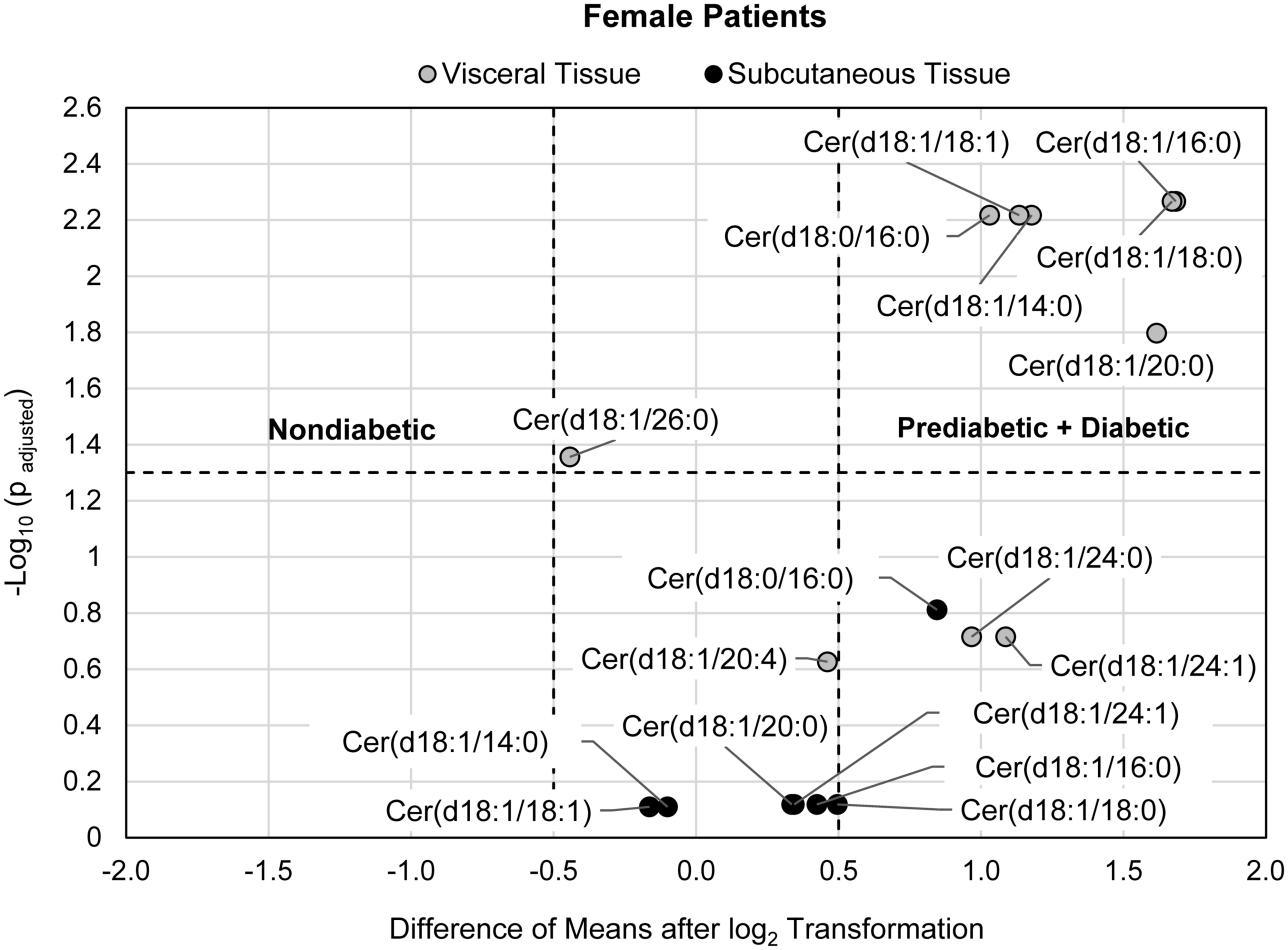
Volcano plot visualizing the difference of means of log_2_ transformed data of the prediabetic/diabetic group with the nondiabetic group for each tissue type (subcutaneous and visceral) for female patients over P‐values of 10 ceramide/dihydroceramide species in visceral tissue from 13 nondiabetic patients and 15 insulin resistant patients. For analysis of subcutaneous tissue, 7 ceramide/dihydroceramide species were evaluated from 10 nondiabetic patients and 14 insulin resistant patients. The P‐values were calculated by a Welch's t‐test with false discovery rate (FDR) correction. The dotted horizontal line indicates a statistical threshold of *p* = .05, and the dotted vertical lines mark a threshold value of an absolute difference in means of 0.5. The figure shows the mean concentration of ceramide species in visceral tissue to be higher in the insulin resistant group

**FIGURE 6:**
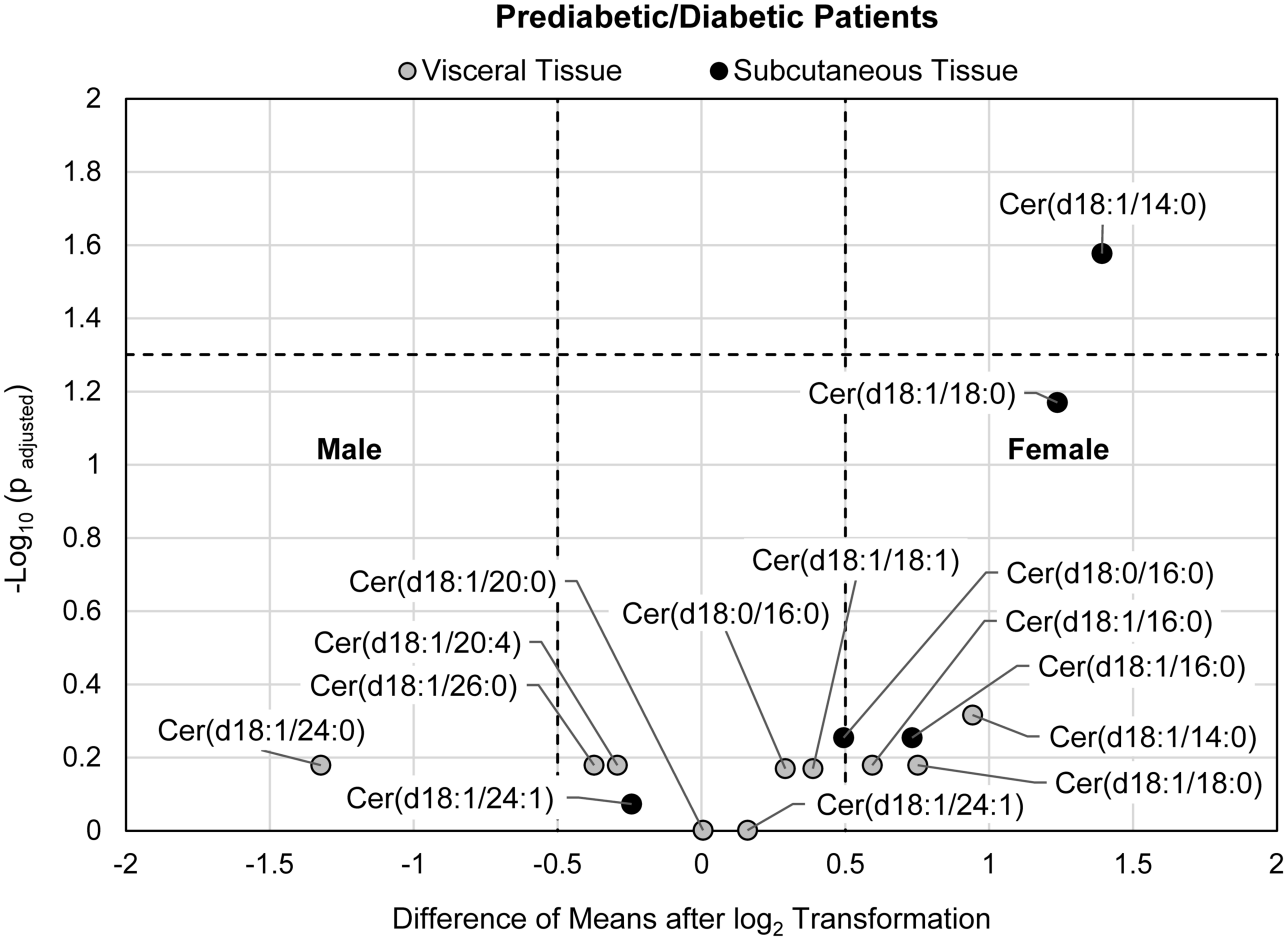
Volcano plot visualizing the difference of means of log_2_ transformed data of female patients with male patients in the insulin resistant group for each tissue type (subcutaneous and visceral) over P‐values of 10 ceramide/dihydroceramide species in visceral tissue from 7 male patients and 15 female patients. For analysis of subcutaneous tissue, 5 ceramide/dihydroceramide species were evaluated from 6 male patients and 14 female patients. The P‐values were calculated by a Welch's t‐test with false discovery rate (FDR) correction. The dotted horizontal line indicates a statistical threshold of *p* = .05, and the dotted vertical lines mark a threshold value of an absolute difference in means of 0.5. The figure shows the mean concentration of ceramide Cer(d18:1/14:0) in subcutaneous tissue to be higher in the female subjects

A separate unpaired analysis by patient group was conducted for visceral and subcutaneous tissue. To examine ceramide/dihydroceramide content in tissue between patient groups, the difference of the means of the log_2_‐transformed data was evaluated. Figures 4, 5, 6 show results for all patients (combined groups of male and female subjects), female patients only, and male vs female subjects, respectively.

For all patients, a statistically significant difference of the means of the log_2_‐transformed concentration for each of the seven ceramide/dihydroceramide species evaluated in subcutaneous tissue was not evident between nondiabetic (11 patients, with 10 female subjects and 1 male subject) and insulin‐resistant subjects (20 patients, with 14 female subjects and 6 male subjects) (Figure 4). Examination of the difference of means between patient groups in visceral tissue revealed that 7 out of 10 ceramides/dihydroceramides species were higher in the prediabetic/diabetic group (22 patients, with 15 female subjects and 7 male subjects) compared with the nondiabetic group (14 patients, with 13 female subjects and 1 male subject) (*P*_adjusted_ < .05, difference of means >0.5).

When considering only female patients, 6 out of 10 ceramides/dihydroceramides species evaluated in visceral tissue (Figure 5) are shown to be higher in the insulin‐resistant group (n = 15) compared with the nondiabetic group (n = 13; *P*_adjusted_ < .05, difference of means >0.5), while a statistically significant difference of the means of the log_2_‐transformed concentration for each of the seven ceramide/dihydroceramide species evaluated in subcutaneous tissue between patient groups was not evident.

Figure 6 shows the difference of means of log_2_‐transformed data between female and male patients in the insulin‐resistant group for each tissue type (subcutaneous and visceral). Ten ceramide/dihydroceramide species were examined in visceral tissue (7 male subjects and 15 female patients), while five species were evaluated in subcutaneous tissue (6 male patients and 14 female patients). Results indicated Cer(d18:1/14:0) to be higher in subcutaneous tissue of insulin‐resistant females compared with insulin‐resistant males (*P*_adjusted_ < .05, difference of means >0.5).

Unpaired analyses of ceramide/dihydroceramide species between insulin‐resistant AA (with visceral tissue samples from 17 patients, 4 male and 13 female, and subcutaneous tissue samples from 16 subjects, 4 male and 12 female) and insulin‐resistant Caucasian patients (with visceral tissue samples from 5 patients, 3 male and 2 female, and subcutaneous tissue samples from 4 subjects, 2 male and 2 female) were evaluated. After *P* value correction, a statistically significant difference of the means of the log_2_‐transformed concentration for each of the 10 ceramide/dihydroceramide species evaluated in visceral tissue and for the 2 species, Cer(d18:1/14:0) and Cer(d18:1/16:0), evaluated in subcutaneous tissue were not evident (ie, the data do not provide enough evidence against the null hypotheses). Adjusted and unadjusted *P* values are provided in Supplementary Tables S3 and S4.

## 4 DISCUSSION

Adipose tissue is the largest primary energy storage depot in the body, where fat is stored in the form of triacylglycerols both in the subcutaneous and visceral adipose tissue. In obesity, where there is an excessive energy depot, there is a spillover of lipid metabolites into circulation. This in turn causes ectopic fat deposition in the liver and skeletal muscle that results in lipotoxicity. In the subsequent spectrum of cellular dysfunction, ceramides have been identified as the key contributors to metabolic disruption that leads to dysregulation of glucose metabolism, TG synthesis, and apoptosis. Accumulation of ceramides and their metabolites is implicated in the development of IR as well as obesity‐related myocardial injury,^29^, ^34^ and elevated plasma ceramide concentration is a strong predictor of cardiovascular events^35^ and type 2 diabetes.^36^, ^37^ Ceramide levels can be reduced and insulin signaling can be significantly improved by the inhibition of ceramide biosynthesis enzymes with pharmacologic agents or small interfering RNA, or through overexpression of ceramide‐metabolizing acid ceramidase.^8^, ^10^ However, the precise mechanism by which ceramides cause the metabolic abnormalities is unclear.^8^, ^10^

Given ceramides modulate adipose tissue function, the aim of this preliminary study is to identify ceramide species by LC‐MS that accumulate in central fat in relation to status of diabetes and gender for subjects with BMI ≥40 kg/m^2^ or > 35 kg/m^2^ with comorbidity. To characterize ceramides/dihydroceramides in tissue, two types of analysis were performed on log_2_‐transformed data. Paired samples collected from different adipose tissue compartments of nondiabetic and insulin‐resistant patients were independently analyzed to examine the distribution of ceramide species between visceral and subcutaneous tissue. Within‐tissue analysis was performed to unveil differences in ceramide content between nondiabetic and insulin‐resistant patients for each tissue type (visceral and subcutaneous). Visceral tissue is in proximity of internal organs and is more cellular and vascular and contains a larger number of immune and inflammatory cells compared with subcutaneous tissue.^29^ Adipocytes in visceral adipose tissue are more metabolically active and have a greater tendency to exhibit IR than adipocytes in subcutaneous adipose tissue.^29^ Our findings indicate a potential association of several ceramide species with IR and gender‐dependent accumulation of ceramide species in visceral tissue with respect to subcutaneous tissue.

The analyses all patients (comprising both genders, with the majority being female) showed ceramide species that exhibited differential abundance in visceral tissue of insulin‐resistant subjects also had higher mean levels compared with nondiabetic patients, with the exception of Cer(d18:1/24:1) and Cer(d18:1/14:0). While ceramide Cer(d18/1:24:1) exhibited differential accumulation in visceral tissue, unpaired analysis showed the difference of means between insulin‐resistant and nondiabetic subjects was not large enough to be statistically significant. Conversely, unpaired analysis of Cer(d18:1/14:0) showed an elevated mean level in visceral tissue of insulin‐resistant patients compared with nondiabetic subjects, while paired analysis of visceral to subcutaneous tissue showed no significant differential accumulation. This trend was also observed in the analysis of female only subjects. Ceramide species Cer(d18:1/14:0) has been associated with insulin sensitivity. Kasumov et al found that improved insulin sensitivity, after a 12‐week exercise training program, was linked to reduced plasma Cer(d18:1/14:0) in subjects with obesity and type 2 diabetes.^38^

When considering only female patients, Cer(d18:1/16:0), Cer(d18:1/18:0), Cer(d18:1/18:1), Cer(d18:1/20:0), Cer(d18:1/24:1), and DhCer(d18:0/16:0) exhibited differential accumulation in visceral tissue of insulin‐resistant patients and higher mean levels of Cer(d18:1/14:0), Cer(d18:1/16:0), Cer(d18:1/18:0), Cer(d18:1/18:1), Cer(d18:1/20:0), and DhCer(d18:0/16:0) in insulin‐resistant subjects compared with nondiabetic patients. In a small cohort study, Choromańska et al. found in females subjects with obesity Cer(d18:1/16:0), Cer(d18:1/18:0), Cer(d18:1/20:0), Cer(d18:1/22:0), and Cer(d18:1/24:1) to be higher in visceral tissue in relation to subcutaneous tissue in subjects with metabolic syndrome and higher levels of Cer(d18:1/16:0) and Cer(d18:1/18:0) in females with metabolic syndrome compared with females without metabolic syndrome.^21^ The accumulation of Cer(d18:1/16:0) and Cer(d18:1/18:0) can induce IR. Cer(d18:1/18:0) has been found to impair systemic glucose homeostasis in skeletal muscle, while Cer(d18:1/16:0) has been found to impair insulin action in brown adipose tissue and liver.^39^

To further examine the relationship of ceramide species with gender, paired analysis of visceral to subcutaneous tissue for male subjects was conducted. Owing to the distribution of males in the nondiabetic group (n = 1), only insulin‐resistant patients were analyzed. Comparison of the results with those obtained for insulin‐resistant female subjects show that of the four ceramides in common, insulin‐resistant female patients, but not male, exhibited differential accumulation of Cer(d18:1/18:0), Cer(d18:1/24:1), and Cer(d18:1/16:0) in visceral tissue. To examine differences in mean levels of ceramide species between insulin‐resistant males and insulin‐resistant females, unpaired analysis was conducted and revealed higher content of Cer(d18:1/14:0) in subcutaneous tissue of females compared with males. In a small cohort study, Blachnio‐Zabielska et al. investigated ceramide metabolism in human subcutaneous adipose tissue of lean male and female subjects and those with obesity and found a number of ceramide subspecies that displayed sex differences.^22^ They also revealed for females only a negative correlation between Cer(d18:1/16:0) ceramide and plasma adiponectin and a positive correlation between total ceramide content and homeostatic model assessment of insulin resistance (HOMA‐IR).^22^ Taken together, these results suggest that gender may influence the differential expression of ceramide species between visceral and subcutaneous tissue and ceramide content in relation to the status of diabetes.

The gender‐specific role of sphingolipids in obesity‐related metabolic dysfunctions is unclear.^40^ Studies that take into account differences in sphingolipids levels between genders are still evolving.^40^ While both visceral and subcutaneous adipose tissue increase with increasing weight for both genders, there are differences in fat distribution between females and males.^41^ In general, females present lower levels of visceral abdominal adiposity than males,^41^ where abdominal visceral adipose tissue has been shown to correlate with sex differences for cardiovascular risk.^41^ Factors such as age and hormone levels (estrogen and testosterone) that affect visceral adiposity^41^ in addition to sex differences that co‐occur with obesity (HDL cholesterol, plasma insulin, glucose, and TG^41^) may correlate with the accumulation of gender‐specific ceramides and may prove beneficial when profiling diabetic risk.

Given that AA are at increased risk for the development of diabetes, it is important to understand ceramide biosynthesis and metabolism in this population compared to Caucasians. Due to study limitation (one Caucasian patient in the nondiabetic group), only insulin‐resistant subjects were evaluated. For each ceramide species, unpaired analysis between insulin‐resistant AA and insulin‐resistant Caucasian patients did not reveal statically significant differences in mean levels. In other words, the null hypothesis of equal means could not be rejected. Unlike the classical two‐sample *t* test, the Welch *t* test does not assume that the two populations sampled have equal variance. In simulation, for the case of unequal sample size (for n ≤ 20) and unequal variance, the simulated levels of significance for the Welch test closely matched the target level of significance (type I effort rate: the probability of rejecting the null hypothesis when it is actually true).^42^ Hence, it is concluded that the Welch *t* test is reliable when data provide enough evidence against the null hypothesis. However, for small sample sizes, as with this study, the power of the Welch *t* test may not be adequate to detect a difference between means when a difference truly exists (leading to type II error). In other words, while significant differences between some groups in this work were not noted, differences between the populations may exist.

This preliminary work highlights the need for a larger cohort study to examine further the relationship of age, race, and gender with ceramide content in subcutaneous and visceral adipose tissue relative to the status of diabetes. Correlating these finding with biological function could aid in the development of therapeutic intervention.

## 5 CONCLUSION

The analysis of ceramide/dihydroceramide species is important for developing a better understanding of biological processes underlying type 2 diabetes, metabolic syndrome, and obesity and how to target them for better patient outcomes. Our results have revealed, for subjects with BMI ≥40 kg/m^2^ or > 35 kg/m^2^ with comorbidity, significantly increased ceramide levels in visceral tissue of patients with diabetes and prediabetes compared with nondiabetic subjects. We have determined that several ceramide species are differentially expressed in insulin‐resistant females compared to insulin‐resistant males.

## CONFLICT OF INTEREST

The authors declared no conflict of interest.

## AUTHOR CONTRIBUTIONS

All authors were involved in writing the paper and had final approval of the submitted and published versions. Additionally, tissue procurement was done by Michael Wood and Berhane Seyoum, tissue processing and extraction by Stephanie Tucker, lipidomic analysis by Krishnaroa Maddipati, and statistical analyses by Michelle Brusatori, S. Kiran Koya, and Gregory Auner.

## Supporting information


**Appendix S1**: Supporting Information
