# Supplementary material for: Ceramide changes in abdominal subcutaneous and visceral adipose tissue among diabetic and nondiabetic patients
Source: J Diabetes. 2022 Apr 25;14(4):271–81. doi: 10.1111/1753-0407.13262 (PMC9060146; doi:10.1111/1753-0407.13262)
Supplement: Supplementary file 1 — Appendix S1: Supporting Information [file JDB-14-271-s001.docx]

**SUPPLEMENTARY MATERIAL**

Samples of visceral and abdominal wall subcutaneous adipose tissue (n=36, n=31, respectively) were taken during laparoscopic surgery from 36 patients with BMI >35kg/m² with ≥1 existing comorbidity or BMI ≥40 kg/m² and a history of prolonged previous attempts of weight loss by other means. For each patient category (all patients, female, male, African American and Caucasian), the mean concentration +/- the standard deviation of the original data and of the log_2_ transformed concentration of each ceramide/dihydroceramide species evaluated in this work is presented in **Tables S1** and **S2**. Variables with less than 37% missingness, within and between tissue type, for each subgroup were retained for analysis.

**Visceral Tissue**

**Table S1**: Mean ceramide/dihydroceramide concentration in visceral tissue for each patient category.

| **Visceral Tissue** | | | | | |
| --- | --- | --- | --- | --- | --- |
|  | **Cer(d18:1/14:0)** | **Cer(d18:1/16:0)** | **DhCer(d18:0/16:0)** | **Cer(d18:1/18:0)** | **Cer(d18:1/18:1)** |
| All Patients  Nondiabetic | n = 14 patients | n = 14 patients | n = 14 patients | n = 14 patients | n = 14 patients |
| Mean (ng/mg protein) | 0.127 +/- 0.067 | 0.867 +/- 0.421 | 0.047 +/- 0.022 | 0.079 +/- 0.064 | 0.032 +/- 0.013 |
| Mean of Log_2_ transformed data | -3.180 +/- 0.821 | -0.397 +/- 0.821 | -4.386 +/- 0.515 | -4.055 +/- 1.095 | -5.098 +/- 0.669 |
| % Missing data | 0 % | 0 % | 29 % (36 % after outlier removal) | 0 % | 21 % |
| All Patients  Prediabetic + Diabetic | n = 22 patients | n = 22 patients | n = 22 patients | n = 22 patients | n = 22 patients |
| Mean (ng/mg protein) | 0.245 +/- 0.193 | 3.057 +/- 2.377 | 0.105 +/- 0.060 | 0.228 +/- 0.195 | 0.074 +/- 0.044 |
| Mean of Log_2_ transformed data | -2.426 +/- 1.117 | 1.047 +/- 1.454 | -3.549 +/- 1.063 | -2.685 +/- 1.374 | -4.019+/- 0.964 |
| % Missing data | 0 % | 0 % | 14 % | 5 % | 14 % |
| Female  Nondiabetic | n = 13 patients | n = 13 patients | n = 13 patients | n = 13 patients | n = 13 patients |
| Mean (ng/mg protein) | 0.127 +/- 0.070 | 0.842 +/- 0.428 | 0.049 +/- 0.023 | 0.078 +/- 0.067 | 0.033 +/- 0.014 |
| Mean of Log_2_ transformed data | -3.196 +/- 0.852 | -0.447 +/- 0.832 | -4.331 +/- 1.122 | -4.105 +/- 1.095 | -5.051 +/- 0.686 |
| % Missing data | 0 % | 0 % | 31 % | 0 % | 23 % |
| Female  Prediabetic + Diabetic | n = 15 patients | n = 15 patients | n = 15 patients | n = 15 patients | n = 15 patients |
| Mean (ng/mg protein) | 0.288 +/- 0.214 | 3.422 +/- 2.460 | 0.108 +/- 0.065 | 0.245 +/- 0.184 | 0.079 +/- 0.047 |
| Mean of Log_2_ transformed data | -2.019 +/- 1.007 | 1.236 +/- 1.492 | -3.301 +/- 0.781 | -2.434 +/- 1.211 | -3.917 +/- 0.967 |
| % Missing data | 0 % (7 % after outlier removal) | 0 % | 7 % (13 % after outlier removal) | 7 % | 7 % |
| Male  Nondiabetic | n = 1 patient | n = 1 patient | n = 1 patient | n = 1 patient | n = 1 patient |
| Amount (ng/mg protein) | 0.128 | 1.185 | 0.035 | 0.094 | 0.021 |
| Log_2_ transformed data | -2.969 | 0.245 | -4.823 | -3.404 | -5.568 |
| Male  Prediabetic + Diabetic | n = 7 patients | n = 7 patients | n = 7 patients | n = 7 patients | n = 7 patients |
| Mean (ng/mg protein) | 0.152 +/- 0.097 | 2.275 +/- 2.146 | 0.095 +/- 0.051 | 0.192 +/- 0.226 | 0.060 +/- 0.034 |
| Mean of Log_2_ transformed data | -2.961 +/- 0.899 | 0.642 +/- 1.386 | -3.592 +/- 0.886 | -3.186 +/- 1.635 | -4.304 +/- 1.002 |
| % Missing data | 0 % | 0 % | 29 % | 0 % | 29 % |
| African American  Nondiabetic | n = 13 patients | n = 13 patients | n = 13 patients | n = 13 patients | n = 13 patients |
| Mean (ng/mg protein) | 0.132 +/- 0.067 | 0.901 +/- 0.417 | 0.047 +/- 0.023 | 0.078 +/- 0.067 | 0.032 +/- 0.013 |
| Mean of Log_2_ transformed data | -3.125 +/- 0.827 | -0.331 +/- 0.814 | -4.570 +/- 0.753 | -4.101 +/- 1.125 | -5.098 +/- 0.669 |
| % Missing data | 0 % | 0 % | 31 % | 0 % | 15 % |
| African American  Prediabetic + Diabetic | n = 17 patients | n = 17 patients | n = 17 patients | n = 17 patients | n = 17 patients |
| Mean (ng/mg protein) | 0.260 +/- 0.212 | 3.029 +/- 2.620 | 0.096 +/- 0.061 | 0.173 +/- 0.122 | 0.072 +/- 0.049 |
| Mean of Log_2_ transformed data | -2.386 +/- 1.210 | 0.907 +/- 1.599 | -3.701 +/- 1.115 | -2.974 +/- 1.264 | -4.130 +/- 1.059 |
| % Missing data | 0 % | 0 % | 18 % | 6 % | 12 % |
| Caucasian  Nondiabetic | n = 1 patient | n = 1 patient | n = 1 patient | n = 1 patient | n = 1 patient |
| Amount (ng/mg protein) | 0.067 | 0.418 | 0.048 | 0.091 | NA |
| Log_2_ transformed data | -3.891 | -1.260 | -4.380 | -3.457 | NA |
| Caucasian  Prediabetic + Diabetic | n = 5 patients | n = 5 patients | n = 5 patients | n = 5 patients | n = 5 patients |
| Mean (ng/mg protein) | 0.191 +/- 0.108 | 3.153 +/- 1.481 | 0.129 +/- 0.057 | 0.402 +/- 0.290 | 0.083 +/- 0.013 |
| Mean of Log_2_ transformed data | -2.564 +/- 0.812 | 1.524 +/- 0.705 | -3.126 +/- 0.861 | -1.760 +/- 1.428 | -3.603 +/- 0.233 |
| % Missing data | 0 % | 0 % | 0 % | 0 % | 20 % |

| **Visceral Tissue Continued** | | | | | |
| --- | --- | --- | --- | --- | --- |
|  | **Cer(d18:1/20:0)** | **Cer(d18:1/20:4)** | **Cer(d18:1/24:0)** | **Cer(d18:1/24:1)** | **Cer(d18:1/26:0)** |
| All Patients  Nondiabetic | n = 14 patients | n = 14 patients | n = 14 patients | n = 14 patients | n = 14 patients |
| Mean (ng/mg protein) | 0.095 +/- 0.144 | 0.013 +/- 0.007 | 0.064 +/- 0.092 | 0.170 +/- 0.266 | 0.009 +/- 0.003 |
| Mean of Log_2_ transformed data | -4.641 +/- 1.223 | -6.517 +/- 0.940 | -4.982 +/- 1.705 | -3.818 +/- 1.871 | -6.735 +/- 0.356 |
| % Missing data | 7 % (14 % after outlier removal) | 29 % | 0 % | 14 % | 36% (43 % after outlier removal) |
| All Patients  Prediabetic + Diabetic | n = 22 patients | n = 22 patients | n = 22 patients | n = 22 patients | n = 22 patients |
| Mean (ng/mg protein) | 0.232 +/- 0.220 | 0.018 +/- 0.009 | 0.171 +/- 0.200 | 0.392 +/- 0.515 | 0.009 +/- 0.004 |
| Mean of Log_2_ transformed data | -2.828 +/- 1.634 | -5.971 +/- 0.726 | -3.519 +/- 1.768 | -2.688 +/- 2.187 | -6.961 +/- 0.596 |
| % Missing data | 18 % | 23 % | 23 % | 9 % | 32 % |
| Female  Nondiabetic | n = 13 patients | n = 13 patients | n = 13 patients | n = 13 patients | n = 13 patients |
| Mean (ng/mg protein) | 0.100 +/- 0.149 | 0.013 +/- 0.007 | 0.068 +/- 0.094 | 0.182 +/- 0.275 | 0.009 +/- 0.003 |
| Mean of Log_2_ transformed data | -4.283 +/- 1.614 | -6.517 +/- 0.940 | -4.875 +/- 1.726 | -3.718 +/- 1.928 | -6.735 +/- 0.356 |
| % Missing data | 8 % | 23 % | 0 % | 15 % | 31 % |
| Female  Prediabetic + Diabetic | n = 15 patients | n = 15 patients | n = 15 patients | n = 15 patients | n = 15 patients |
| Mean (ng/mg protein) | 0.186 +/- 0.156 | 0.017 +/- 0.010 | 0.119 +/- 0.138 | 0.322 +/- 0.410 | 0.008 +/- 0.004 |
| Mean of Log_2_ transformed data | -2.667 +/- 1.033 | -6.057 +/- 0.796 | -3.908 +/- 1.611 | -2.632 +/- 1.821 | -7.178 +/- 0.410 |
| % Missing data | 20% (27 % after outlier removal) | 20 % | 20 % | 13 % | 33% |
| Male  Nondiabetic | n = 1 patient | n = 1 patient | n = 1 patient | n = 1 patient | n = 1 patient |
| Amount (ng/mg protein) | 0.027 | NA | 0.012 | 0.033 | NA |
| Log2 transformed data | -5.190 | NA | -6.367 | -4.918 | NA |
| Male  Prediabetic + Diabetic | n = 6 patients | n = 6 patients | n = 6 patients | n = 6 patients | n = 6 patients |
| Mean (ng/mg protein) | 0.326 +/- 0.309 | 0.019 +/-0.007 | 0.295 +/- 0.283 | 0.523 +/- 0.688 | 0.010 +/- 0.004 |
| Mean of Log_2_ transformed data | -2.672 +/- 2.321 | -5.764 +/- 0.537 | -2.585 +/- 1.953 | -2.792 +/- 2.915 | -6.805 +/- 0.655 |
| % Missing data | 14 % | 29 % | 29 % | 0 % | 29 % |
| African American  Nondiabetic | n = 13 patients | n = 13 patients | n = 13 patients | n = 13 patients | n = 13 patients |
| Mean (ng/mg protein) | 0.090 +/- 0.150 | 0.013 +/- 0.007 | 0.061 +/- 0.095 | 0.168 +/- 0.278 | 0.009 +/- 0.003 |
| Mean of Log_2_ transformed data | -4.483 +/- 1.559 | -6.472 +/- 0.985 | -5.118 +/- 1.694 | -3.949 +/- 1.904 | -6.837 +/- 0.521 |
| % Missing data | 8 % | 31 % | 0 % | 15 % | 39 % |
| African American  Prediabetic + Diabetic | n = 17 patients | n = 17 patients | n = 17 patients | n = 17 patients | n = 17 patients |
| Mean (ng/mg protein) | 0.151 +/- 0.129 | 0.017 +/- 0.010 | 0.099 +/- 0.106 | 0.211 +/- 0.261 | 0.008 +/- 0.004 |
| Mean of Log_2_ transformed data | -3.334 +/- 1.516 | -6.039 +/- 0.741 | -3.981 +/- 1.403 | -3.323 +/- 1.948 | -6.981 +/- 0.543 |
| % Missing data | 24 % | 24 % | 29 % | 12 % | 35 % |
| Caucasian  Nondiabetic | n = 1 patient | n = 1 patient | n = 1 patient | n = 1 patient | n = 1 patient |
| Amount (ng/mg protein) | 0.145 | 0.008 | 0.108 | 0.193 | 0.008 |
| Log2 transformed data | -2.782 | -6.925 | -3.211 | -2.376 | -7.003 |
| Caucasian  Prediabetic + Diabetic | n = 5 patients | n = 5 patients | n = 5 patients | n = 5 patients | n = 5 patients |
| Mean (ng/mg protein) | 0.443 +/- 0.282 | 0.020 +/- 0.009 | 0.343 +/- 0.276 | 0.938 +/- 0.726 | 0.009 +/- 0.005 |
| Mean of Log_2_ transformed data | -1.513 +/- 1.207 | -5.748 +/- 0.723 | -2.409 +/- 2.215 | -0.782 +/- 1.839 | -6.906 +/- 0.819 |
| % Missing data | 0 % | 20 % | 0 % | 0 % | 20 % |

**Subcutaneous Tissue**

**Table S2**: Mean ceramide/dihydroceramide concentration in subcutaneous tissue for each patient category.

| **Subcutaneous Tissue** | | | | | |
| --- | --- | --- | --- | --- | --- |
|  | **Cer(d18:1/14:0)** | **Cer(d18:1/16:0)** | **DhCer(d18:0/16:0)** | **Cer(d18:1/18:0)** | **Cer(d18:1/18:1)** |
| All Patients  Nondiabetic | n = 11 patients | n = 11 patients | n = 11 patients | n = 11 patients | n = 11 patients |
| Mean (ng/mg protein) | 0.226 +/- 0.180 | 1.839 +/- 2.516 | 0.043 +/- 0.032 | 0.093 +/- 0.081 | 0.047 +/- 0.028 |
| Mean of Log_2_ transformed data | -2.290 +/- 0.576 | -0.017 +/- 1.725 | -5.006 +/- 0.645 | -3.928 +/- 1.280 | -4.657 +/- 0.936 |
| % Missing data | 0% (27 % after outlier removal) | 0 % | 18% (27 % after outlier removal) | 9 % | 27 % |
| All Patients  Prediabetic + Diabetic | n = 20 patients | n = 20 patients | n = 20 patients | n = 20 patients | n = 20 patients |
| Mean (ng/mg protein) | 0.186 +/- 0.137 | 1.698 +/- 1.233 | 0.056 +/- 0.029 | 0.114 +/- 0.104 | 0.042 +/- 0.021 |
| Mean of Log_2_ transformed data | -2.815 +/- 1.140 | 0.261 +/- 1.398 | -4.345 +/- 0.812 | -3.593 +/- 1.192 | -4.780 +/- 0.815 |
| % Missing data | 0 % | 5 % | 20 % | 15 % | 25 % |
| Female  Nondiabetic | n = 10 patients | n = 10 patients | n = 10 patients | n = 10 patients | n = 10 patients |
| Mean (ng/mg protein) | 0.232 +/- 0.188 | 1.951 +/- 2.622 | 0.044 +/- 0.034 | 0.098 +/- 0.084 | 0.050 +/- 0.029 |
| Mean of Log_2_ transformed data | -2.038 +/- 0.796 | 0.030 +/- 1.811 | -5.067 +/- 0.671 | -3.868 +/- 1.343 | -4.545 +/- 0.952 |
| % Missing data | 0 % 20% after outlier removal) | 0 % | 20 % (30% after outlier removal) | 10 % | 30 % |
| Female  Prediabetic + Diabetic | n = 14 patients | n = 14 patients | n = 14 patients | n = 14 patients | n = 14 patients |
| Mean (ng/mg protein) | 0.223 +/- 0.145 | 1.899 +/- 1.300 | 0.060 +/- 0.029 | 0.129 +/- 0.113 | 0.043 +/- 0.020 |
| Mean of Log_2_ transformed data | -2.138 +/- 0.693 | 0.454 +/- 1.392 | -4.222 +/- 0.713 | -3.372 +/- 1.155 | -4.709 +/- 0.753 |
| % Missing data | 0 % (14% after outlier removal) | 0 % | 14 % | 14 % | 14 % |
| Male  Nondiabetic | n = 1 patient | n = 1 patient | n = 1 patient | n = 1 patient | n = 1 patient |
| Amount (ng/mg protein) | 0.159 | 0.715 | 0.042 | 0.045 | 0.023 |
| Log_2_ transformed data | -2.651 | -0.484 | -4.576 | -4.472 | -5.440 |
| Male  Prediabetic + Diabetic | n = 6 patients | n = 6 patients | n = 6 patients | n = 6 patients | n = 6 patients |
| Mean (ng/mg protein) | 0.099 +/- 0.058 | 1.135 +/- 0.904 | 0.047 +/- 0.033 | 0.079 +/- 0.081 | 0.037 +/- 0.028 |
| Mean of Log_2_ transformed data | -3.530 +/- 0.793 | -0.278 +/- 1.415 | -4.716 +/- 1.090 | -4.609 +/- 0.670 | -5.063 +/- 1.176 |
| % Missing data | 0 % | 17 % | 33 % | 17 % | 50 % |
| African American  Nondiabetic | n = 10 patients | n = 10 patients | n = 10 patients | n = 10 patients | n = 10 patients |
| Mean (ng/mg protein) | 0.204 +/- 0.173 | 1.850 +/- 2.651 | 0.045 +/- 0.034 | 0.093 +/- 0.086 | 0.046 +/- 0.030 |
| Mean of Log_2_ transformed data | -2.745 +/- 1.281 | -0.098 +/- 1.796 | -4.756 +/- 0.945 | -3.983 +/- 1.346 | -4.734 +/- 0.984 |
| % Missing data | 0 % | 0 % | 20 % | 10 % | 30 % |
| African American  Prediabetic + Diabetic | n = 16 patients | n = 16 patients | n = 16 patients | n = 16 patients | n = 16 patients |
| Mean (ng/mg protein) | 0.210 +/- 0.141 | 1.884 +/- 1.241 | 0.059 +/- 0.030 | 0.113 +/- 0.105 | 0.045 +/- 0.021 |
| Mean of Log_2_ transformed data | -2.595 +/- 1.120 | 0.473 +/- 1.354 | -4.262 +/- 0.791 | -3.577 +/- 1.134 | -4.676 +/- 0.817 |
| % Missing data | 0 % | 0 % | 13 % | 6 % | 19 % |
| Caucasian  Nondiabetic | n = 1 patient | n = 1 patient | n = 1 patient | n = 1 patient | n = 1 patient |
| Amount (ng/mg protein) | 0.447 | 1.732 | 0.030 | 0.093 | 0.057 |
| Log_2_ transformed data | -1.160 | 0.792 | -5.045 | -3.433 | -4.122 |
| Caucasian  Prediabetic + Diabetic | n = 4 patients | n = 4 patients | n = 4 patients | n = 4 patients | n = 4 patients |
| Mean (ng/mg protein) | 0.086 +/- 0.045 | 0.704 +/- 0.612 | 0.037 +/- 0.023 | 0.123 +/- 0.137 | 0.023 +/- 0.008 |
| Mean of Log_2_ transformed data | -3.697 +/- 0.812 | -0.872 +/- 1.248 | -4.930 +/- 0.982 | -3.713 +/- 2.165 | -5.456 +/- 0.490 |
| % Missing data | 0 % | 25 % | 50 % | 50 % | 50 % |

| **Subcutaneous Tissue Continued** | | | | | |
| --- | --- | --- | --- | --- | --- |
|  | **Cer(d18:1/20:0)** | **Cer(d18:1/20:4)** | **Cer(d18:1/24:0)** | **Cer(d18:1/24:1)** | **Cer(d18:1/26:0)** |
| All Patients  Nondiabetic | n = 11 patients | n = 11 patients | n = 11 patients | n = 11 patients | n = 11 patients |
| Mean (ng/mg protein) | 0.038 +/- 0.021 | 0.008 +/- 0.003 | 0.029 +/- 0.016 | 0.054 +/- 0.047 | 0.010 +/- 0.003 |
| Mean of Log_2_ transformed data | -4.927 +/- 0.843 | -6.959 +/- 0.480 | -5.255 +/- 0.709 | -4.851 +/- 0.522 | -6.640 +/- 0.344 |
| % Missing data | 27 % | 55 % | 27 % | 36% (45 % after outlier removal) | 64 % |
| All Patients  Prediabetic + Diabetic | n = 20 patients | n = 20 patients | n = 20 patients | n = 20 patients | n = 20 patients |
| Mean (ng/mg protein) | 0.106 +/- 0.181 | 0.022 +/- 0.017 | 0.086 +/- 0.145 | 0.189 +/- 0.466 | 0.009 +/- 0.002 |
| Mean of Log_2_ transformed data | -4.508 +/- 1.194 | -5.630 +/- 0.891 | -4.685 +/- 1.711 | -4.680 +/- 0.922 | -6.788 +/- 0.340 |
| % Missing data | 25 % | 45 % | 45 % | 20 % | 55 % |
| Female  Nondiabetic | n = 10 patients | n = 10 patients | n = 10 patients | n = 10 patients | n = 10 patients |
| Mean (ng/mg protein) | 0.038 +/- 0.021 | 0.008 +/- 0.003 | 0.029 +/- 0.016 | 0.054 +/- 0.047 | 0.010 +/- 0.003 |
| Mean of Log_2_ transformed data | -4.927 +/- 0.843 | -6.959 +/- 0.480 | -5.255 +/- 0.709 | -4.851 +/- 0.522 | -6.632 +/- 0.420 |
| % Missing data | 20 % | 50 % | 20 % | 30 % | 70 % |
| Female  Prediabetic + Diabetic | n = 14 patients | n = 14 patients | n = 14 patients | n = 14 patients | n = 14 patients |
| Mean (ng/mg protein) | 0.110 +/- 0.198 | 0.023 +/- 0.018 | 0.093 +/- 0.168 | 0.206 +/- 0.531 | 0.010 +/- 1 |
| Mean of Log_2_ transformed data | -4.592 +/- 1.066 | -5.824 +/- 1.216 | -5.172 +/- 1.019 | -4.506 +/- 0.884 | -6.716 +/- 0.319 |
| % Missing data | 14 % (21% after outlier removal) | 36 % | 43 % (50% after outlier removal) | 14 % (21% after outlier removal) | 57 % |
| Male  Nondiabetic | n = 1 patient | n = 1 patient | n = 1 patient | n = 1 patient | n = 1 patient |
| Amount (ng/mg protein) | NA | NA | NA | NA | 0.010 |
| Log2 transformed data | NA | NA | NA | NA | -6.666 |
| Male  Prediabetic + Diabetic | n = 6 patients | n = 6 patients | n = 6 patients | n = 6 patients | n = 6 patients |
| Mean (ng/mg protein) | 0.093 +/- 0.110 | 0.017 +/- 0.006 | 0.065 +/- 0.077 | 0.137 +/- 0.218 | 0.008 +/- 0.002 |
| Mean of Log_2_ transformed data | -4.201 +/- 1.845 | -5.929 +/- 0.531 | -4.780 +/- 1.966 | -4.263 +/- 2.234 | -6.932 +/- 0.400 |
| % Missing data | 50 % | 67 % | 50 % | 33 % | 50 % |
| African American  Nondiabetic | n = 10 patients | n = 10 patients | n = 10 patients | n = 10 patients | n = 10 patients |
| Mean (ng/mg protein) | 0.038 +/- 0.023 | 0.008 +/- 0.004 | 0.031 +/- 0.016 | 0.058 +/- 0.049 | 0.010 +/- 0.003 |
| Mean of Log_2_ transformed data | -4.946 +/- 0.909 | -6.969 +/- 0.554 | -5.151 +/- 0.698 | -4.408 +/- 0.965 | -6.640 +/- 0.344 |
| % Missing data | 30 % | 60 % | 30 % | 40 % | 60 % |
| African American  Prediabetic + Diabetic | n = 16 patients | n = 16 patients | n = 16 patients | n = 16 patients | n = 16 patients |
| Mean (ng/mg protein) | 0.104 +/- 0.191 | 0.025 +/- 0.017 | 0.086 +/- 0.158 | 0.180 +/- 0.492 | 0.009 +/- 0.002 |
| Mean of Log_2_ transformed data | -4.284 +/- 1.514 | -5.646 +/- 1.110 | -4.732+/- 1.694 | -4.248 +/- 1.744 | -6.864 +/- 0.350 |
| % Missing data | 19 % | 44 % | 44 % | 13 % | 56 % |
| Caucasian  Nondiabetic | n = 1 patient | n = 1 patient | n = 1 patient | n = 1 patient | n = 1 patient |
| Amount (ng/mg protein) | 0.036 | 0.008 | 0.016 | 0.025 | NA |
| Log2 transformed data | -4.796 | -6.920 | -5.981 | -5.340 | NA |
| Caucasian  Prediabetic + Diabetic | n = 4 patients | n = 4 patients | n = 4 patients | n = 4 patients | n = 4 patients |
| Mean (ng/mg protein) | 0.119 +/- 0.141 | 0.010 +/- 0.004 | 0.083 +/- 0.099 | 0.247 +/- 0.308 | 0.011 +/- 0.001 |
| Mean of Log_2_ transformed data | -3.945 +/- 2.477 | -6.728 +/- 0.600 | -4.473 +/- 2.495 | -3.118 +/- 2.845 | -6.521 +/- 0.068 |
| % Missing data | 50 % | 50 % | 50 % | 50 % | 50 % |

**Tables S3** and **S4** show p-values with and without false discovery rate (FDR) correction in Benjamini–Hochberg procedure. All data were log_2_ transformed prior to analysis. P-values for matched ceramide species in visceral and subcutaneous adipose tissue were calculated by a Paired Student’s t-test, **Table S3**. While p-values for the within tissue analysis were calculated by a Welch’s t-test, **Table S4**.

**Table S3:** p-values calculated for paired visceral to subcutaneous tissue analysis.

| **Prediabetic/Diabetic Patients**  **Matched Pair (visceral to subcutaneous)** | | | | | | | | |
| --- | --- | --- | --- | --- | --- | --- | --- | --- |
| **Ceramides Dihydroceramides** | **All Patients**  (6 ceramides evaluated) | | | **Female**  (7 ceramides evaluated) | | **Male**  (4 ceramides evaluated) | | |
|  | p value | Adj. p value | | p value | Adj. p value | p value | | Adj. p value |
| Cer(d18:1/14:0) | 0.136 | 0.136 | | 0.413 | 0.413 | 0.125 | | 0.380 |
| Cer(d18:1/16:0) | 0.018 | 0.029* | | 0.037 | 0.043* | 0.360 | | 0.400 |
| DhCer(d18:0/16:0) | 0.015 | 0.029* | | 0.024 | 0.043* | - | | - |
| Cer(d18:1/18:0) | 0.008 | 0.029* | | 0.031 | 0.043* | 0.190 | | 0.380 |
| Cer(d18:1/18:1) | 0.024 | 0.029* | | 0.016 | 0.043* | - | | - |
| Cer(d18:1/20:0) | - | - | | 0.002 | 0.017* | - | | - |
| Cer(d18:1/24:1) | 0.020 | 0.029* | | 0.034 | 0.043* | 0.400 | | 0.400 |
| **Nondiabetic Patients**  **Matched Pair (visceral to subcutaneous)** | | | | | | | | |
| **Ceramides** | **All Patients**  (5 ceramides evaluated) | | | | **Female**  (5 ceramides evaluated) | | | |
|  | p value | | Adj. p value | | p value | | Adj. p value | |
| Cer(d18:1/14:0) | 0.138 | | 0.690 | | 0.349 | | 0.884 | |
| Cer(d18:1/16:0) | 0.489 | | 0.884 | | 0.387 | | 0.884 | |
| Cer(d18:1/18:0) | 0.887 | | 0.887 | | 0.922 | | 0.922 | |
| Cer(d18:1/20:0) | 0.530 | | 0.884 | | 0.530 | | 0.884 | |
| Cer(d18:1/24:0) | 0.720 | | 0.887 | | 0.724 | | 0.884 | |
| * Adjusted p value <0.05 | | | | | | | | |

**Table S4**: p-values calculated for within tissue analysis.

| **Visceral Within Tissue Analysis**  **(Nondiabetic vs prediabetic/diabetic)** | | | | | **Visceral**  **Prediabetic/diabetic** | | | |
| --- | --- | --- | --- | --- | --- | --- | --- | --- |
| **Ceramides**  **Dihydroceramides** | **All Patients**  (10 ceramides evaluated) | | **Female**  (10 ceramides evaluated) | | **Male vs Female**  (10 ceramides evaluated) | | **African American vs Caucasian** (10 ceramides evaluate) | |
|  | p value | Adj. p value | p value | Adj. p value | p value | Adj. p value | p value | Adj. p value |
| Cer(d18:1/14:0) | 0.026 | 0.038* | 0.003 | 0.006* | 0.048 | 0.483 | 0.710 | 0.789 |
| Cer(d18:1/16:0) | 0.001 | 0.006* | 0.001 | 0.005* | 0.378 | 0.661 | 0.234 | 0.335 |
| DhCer(d18:0/16:0) | 0.009 | 0.019* | 0.002 | 0.006* | 0.541 | 0.676 | 0.024 | 0.109 |
| Cer(d18:1/18:0) | 0.003 | 0.006* | 0.001 | 0.005* | 0.308 | 0.661 | 0.139 | 0.277 |
| Cer(d18:1/18:1) | 0.001 | 0.006* | 0.003 | 0.006* | 0.480 | 0.676 | 0.094 | 0.235 |
| Cer(d18:1/20:0) | 0.002 | 0.006* | 0.010 | 0.016* | 0.996 | 0.996 | 0.025 | 0.109 |
| Cer(d18:1/20:4) | 0.134 | 0.149 | 0.236 | 0.236 | 0.397 | 0.661 | 0.489 | 0.612 |
| Cer(d18:1/24:0) | 0.027 | 0.038* | 0.161 | 0.192 | 0.227 | 0.661 | 0.198 | 0.329 |
| Cer(d18:1/24:1) | 0.133 | 0.149 | 0.173 | 0.192 | 0.898 | 0.996 | 0.033 | 0.109 |
| Cer(d18:1/26:0) | 0.269 | 0.269 | 0.031 | 0.044* | 0.293 | 0.661 | 0.874 | 0.874 |
| **Subcutaneous Within Tissue Analysis**  **(Nondiabetic vs prediabetic/diabetic)** | | | | | **Subcutaneous**  **Prediabetic/diabetic** | | | |
| **Ceramides**  **Dihydroceramides** | **All Patients**  (7 ceramides evaluated) | | **Female**  (7 ceramides evaluated) | | **Male vs Female**  (5 ceramides evaluated) | | **African American vs Caucasian** (2 ceramides evaluated) | |
|  | p value | Adj. p value | p value | Adj. p value | p value | Adj. p value | p value | Adj. p value |
| Cer(d18:1/14:0) | 0.120 | 0.422 | 0.776 | 0.776 | 0.0053 | 0.026* | 0.065 | 0.130 |
| Cer(d18:1/16:0) | 0.654 | 0.760 | 0.543 | 0.761 | 0.352 | 0.557 | 0.191 | 0.191 |
| DhCer(d18:0/16:0) | 0.045 | 0.312 | 0.020 | 0.154 | 0.445 | 0.557 | - | - |
| Cer(d18:1/18:0) | 0.509 | 0.760 | 0.388 | 0.761 | 0.280 | 0.557 | - | - |
| Cer(d18:1/18:1) | 0.760 | 0.760 | 0.705 | 0.776 | - | - | - | - |
| Cer(d18:1/20:0) | 0.350 | 0.760 | 0.455 | 0.761 | - | - | - | - |
| Cer(d18:1/24:1) | 0.607 | 0.760 | 0.329 | 0.761 | 0.845 | 0.845 | - | - |
| * Adjusted p value <0.05 | | | | | | | | |
